# Effects of DOACs on Mouse Melanoma Metastasis and the Inhibitory Mechanism of Edoxaban, a Factor Xa-Specific DOAC

**DOI:** 10.1055/a-2701-4242

**Published:** 2025-09-30

**Authors:** Keiichi Hiramoto, Taisei Watanabe, Masashi Imai, Koji Suzuki

**Affiliations:** 1 Department of Molecular Pathobiology, Faculty of Pharmaceutical Sciences, Suzuka University of Medical Science, Suzuka City, Mie, Japan

**Keywords:** DOAC, edoxaban, PAR2, IL-6, TGFβ1, B16 melanoma cells, tumor metastasis, C57BL/6j mice

## Abstract

**Introduction:**

Direct oral anticoagulants (DOACs) have been widely used in patients with thromboembolism. We previously reported that among DOACs, edoxaban (EDX), a factor Xa (FXa)-specific DOAC, most effectively inhibited the growth of syngeneic non-metastatic murine colon cancer cells implanted in mice via the protease-activated receptor 2 (PAR2) pathway. This study aimed to analyze the effects and mechanism of action of DOACs targeting thrombin or FXa on the metastasis of murine melanoma B16 cells implanted in mice.

**Materials and Methods:**

B16 cells (10 ^6^ cells in 100 μL) were implanted into the tail vein of 8-week-old female C57BL/6j mice ( *n* = 5 per group), followed by daily oral administration of DOACs targeting thrombin (dabigatran etexilate [DABE], 50 mg/kg body weight [bw]) or FXa (rivaroxaban [RVX], 5 mg/kg bw; EDX, 10 mg/kg bw) for 14 days. The effects on tumor metastasis on day 15 and the inhibitory mechanism of the DOAC with the strongest inhibitory effect were analyzed.

**Results:**

Lung metastasis of B16 cells implanted in mice was significantly suppressed in the following order: EDX > RVX ≥ DABE, compared with the saline-treated group. DOPA (3,4-dihydroxyphenylalanine)-positive cell density was significantly reduced from approximately 1,250 cells/mm ^2^ in the saline group to approximately 600 cells/mm ^2^ (RVX and DABE) and approximately 400 cells/mm ^2^ (EDX; *p* < 0.05 or 0.01). Investigating the inhibitory mechanism of EDX revealed that inflammation-associated factors such as PAR2, interleukin 6 (IL-6), and transforming growth factor β1 (TGFβ1); angiogenic factors such as vascular endothelial growth factor A and angiopoietin-2; and epithelial–mesenchymal transition (EMT)-associated factors such as vimentin and snail, which were increased in the lungs of the saline-treated group, all significantly decreased in the EDX-treated group ( *p* < 0.05 or 0.01). In contrast, intercellular tight junction factors exhibited an opposite trend. EDX also inhibited FXa-dependent production of melanin, IL-6, and TGFβ1 in in vitro cultured B16 cells.

**Conclusion:**

Among the tested DOACs, EDX showed the strongest inhibition of B16 cell metastasis in mice, likely via the suppression of inflammation, angiogenesis, and EMT mediated by the FXa-dependent PAR2 and TGFβ pathways in tumor and surrounding tissue cells.

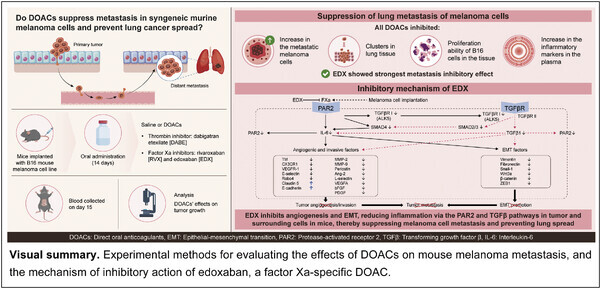

## Introduction

In developed countries with a growing elderly population, the incidence of thrombosis, including arterial, venous, and microvascular thromboses, is rising annually. 1 2 Major causes include cerebral thromboembolism caused by atrial fibrillation, common among older adults, and venous thromboembolism (VTE) owing to surgery for hip or knee joint disease, diabetes, obesity, metabolic disorders, and malignancies. Previously, anticoagulants such as low-molecular-weight heparin (LMWH) and warfarin were used to treat VTE, including in patients with cancer. 3 However, recently, direct oral anticoagulants (DOACs) have largely replaced warfarin for cerebral and pulmonary thromboembolism. 4 5 6 7 The 2022 international clinical practice guidelines for VTE treatment and prophylaxis in patients with cancer recommend LMWH (grade 1A) or DOACs (rivaroxaban [RVX] or apixaban, grade 1B) for primary pharmacological prevention of VTE in outpatients with locally advanced or metastatic pancreatic cancer receiving systemic anticancer therapy, provided bleeding risk is low. 8 Successive randomized clinical trials in patients with cancer and VTE have reported that DOACs are non-inferior to LMWH in preventing VTE recurrence in a 6-month follow-up study, suggesting that DOACs are effective in preventing VTE recurrence in patients with cancer. 9

Approximately one-quarter of VTE cases occur in patients with cancer, including those with leukemia, pancreatic cancer, and lung cancer. 10 Tumor cells activate the hemostatic system in various ways, releasing microparticles containing procoagulant tissue factor (TF) and cancer-associated factor Xa-like protease that can directly activate the coagulation cascade. In addition, tumor cells can stimulate peritumoral cells, such as vascular endothelial cells, epithelial cells, and fibroblasts, present in various organs within the host organ tissues by releasing soluble factors or through direct adhesive contact, further promoting coagulation and systemic inflammation. 11

Anticoagulation therapy for cancer patients with thrombosis can reduce tumor size, and LMWHs specific for factor Xa (FXa), such as dalteparin 12 and nadroparin, 13 significantly improve survival rates in cancer patients compared with treatment without anticoagulation. Although some studies showed positive results, others showed conflicting results. 14 15 Subsequent studies and meta-analyses have shown that LMWHs do not affect the survival of patients with cancer. 16

Recently, we investigated the effects of DOACs (thrombin inhibitor: Dabigatran etexilate [DABE] and FXa inhibitors: RVX and edoxaban [EDX]) on tumor growth in mice implanted with the syngeneic, non-metastatic mouse-derived colon cancer cell line Colon26, and found that among DOACs, EDX significantly inhibited tumor cell growth and induced tumor cell apoptosis via the FXa-protease activated receptor (PAR) 2 pathway, which is activated by coagulation and inflammation in mice. 17

Based on this background, this study aimed to analyze the effectiveness of DOACs against the metastasis of syngeneic metastatic murine melanoma cells implanted in mice, identify the most effective DOAC, and elucidate the mechanism by which this DOAC inhibits melanoma cell metastasis.

## Materials and Methods

### Animals and Melanoma Cells

Allogeneic transplantation studies were performed using specific pathogen-free (SPF) female 8-week-old C57BL/6j mice (SLC, Hamamatsu, Japan). Mice were housed in individual cages in an air-conditioned, SPF-controlled room at 23 ± 1 °C with a 12-hour light/dark cycle (lights on at 08:00 a.m.). Animals had ad libitum access to food and water.

A metastatic mouse melanoma cell line, B16 cells, established from a C57BL/6 mouse tumor 18 and obtained from the Japanese Collection of Research Bioresources Cell Bank (Osaka, Japan), was used at passages 5 to 15. The cells were cultured in Eagle's Minimum Essential Medium (Sigma–Aldrich, Darmstadt, Hesel, Germany) supplemented with 10% serum and L-glutamine. Cells were tested periodically to ensure that they were free from *Mycoplasma* and mouse viruses, or tumor injection. Subconfluent monolayers were harvested after treatment with 1 mM of 0.25% trypsin and 0.02% ethylenediaminetetraacetic acid (Sigma–Aldrich). Trypsinized cells were washed and resuspended in phosphate-buffered saline (PBS; Ca ^2+^ , Mg ^2+^ -free, Sigma–Aldrich).

### Direct Oral Anticoagulants

Thrombin inhibitor (prodrug DABE) and FXa inhibitor (RVX and EDX) were purchased from Nippon Boehringer Ingelheim, Bayer Holding, and Daiichi-Sankyo (Tokyo, Japan), respectively. Each DOAC reagent was dissolved in dimethyl sulfoxide and diluted with saline at different concentrations before being orally administered to mice: DABE (250 mg/mL), RVX (25 mg/mL), or EDX (50 mg/mL), and stored at −30 °C until use. The oral dose of each DOAC was determined based on the dose that ensured sufficient anticoagulation without bleeding symptoms in preclinical studies conducted in mice for the development of DABE, 19 RVX, 20 and EDX. 21 In human clinical studies, oral doses of DABE (3.5–5.0 mg/kg body weight [bw]/day), RVX (0.2–0.3 mg/kg bw/day), and EDX (0.5–1.0 mg/kg bw/day) have been used to prevent thromboembolism. Thus, the doses of DOACs per bw in mice in this study were approximately 10-fold higher than those in humans. These doses are consistent with those used in previous preclinical studies 19 20 21 and those used in our previous study to examine the effects of DOACs on the inhibition of Colon26 cell proliferation. 17

### Evaluation of Direct Oral Anticoagulant Inhibitory Effect on B16 Cell Metastasis in Mice

**Fig. 1:**
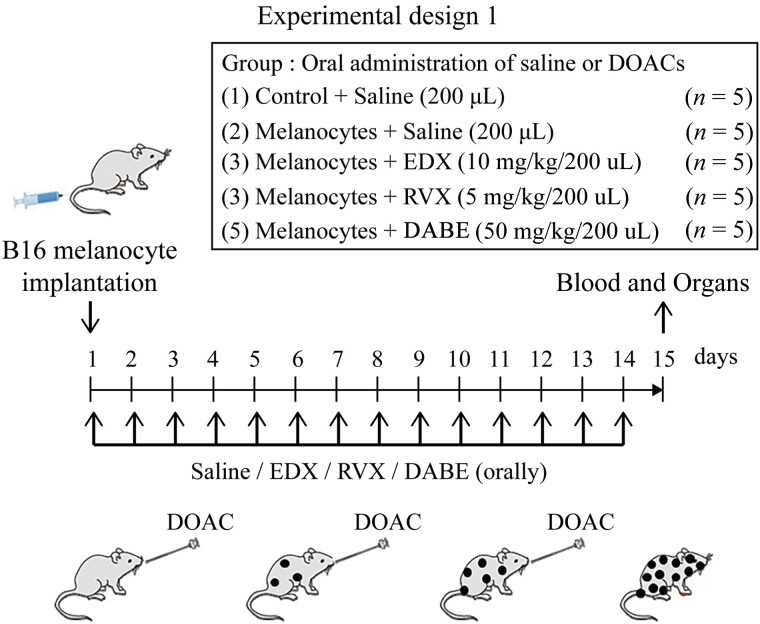
Experimental design 1 evaluates the effects of three DOACs on lung metastasis of B16 cells implanted in 8-week-old female C57BL/6j mice. Cells (100 μL, 1 × 10 ^6^ ) were slowly injected into mice tail veins. Simultaneously, five mice were orally administered 200 μL of saline for 14 days (saline group). In addition, five mice from each group were orally administered 200 μL of edoxaban (EDX: 10 mg/kg bw, EDX group), rivaroxaban (RVX: 5 mg/kg bw, RVX group), or dabigatran etexilate (DABE: 50 mg/kg bw, DABE group) for 14 days after melanoma cell injection. Five untreated mice were used as the control group. On day 15, each mouse was anesthetized, and abdominal surgery was performed to collect heparinized blood and lungs. bw, body weight; DOAC, direct oral anticoagulant; EDX, edoxaban.

To evaluate melanoma cell metastasis in mice, as shown in Fig. 1 , 100 μL of B16 cells (10 ^6^ cells) were implanted via the tail vein of 8-week-old female C57BL/6j mice. From that day, the mice were orally administered 200 μL of saline (saline group) for 14 days. Untreated mice served as a control group. In addition to the control ( *n* = 5) and saline ( *n* = 5) groups, the inhibitory effects of DOACs on melanoma cell metastasis in mice were assessed. Mice were orally administered 200 μL of EDX (10 mg/kg bw, EDX group, *n* = 5), RVX (5 mg/kg bw, RVX group, *n* = 5), or DABE (50 mg/kg bw, DABE group, *n* = 5) for 14 consecutive days. On day 15, mice were anesthetized and underwent abdominal incision, blood was collected from the aorta, and various organs were collected. Blood was mixed with 1/10 volume of 100 IU/mL sodium heparin and centrifuged at 3,000 × *g* at 4 °C for 10 minutes using a cooling centrifuge. The heparinized plasma and organ tissues were stored at −80 °C for later use.

### Organ Tissue Preparation and Staining

Organ tissue samples were isolated and fixed in PBS containing 4% paraformaldehyde (Fujifilm Wako Pure Chemicals, Osaka, Japan). Fixed tissue specimens were embedded in a frozen Tissue-Tek OCT Compound (Sakura Finetek, Tokyo, Japan) and sliced into 5-μm-thick sections. The organ specimens were stained with hematoxylin and eosin (HE) in accordance with established procedures for histological analysis and microscopically evaluated. To identify melanoma cells in the tissue, 3,4-dihydroxyphenylalanine (DOPA)-positive cells, which indicate melanocyte tyrosinase activity, were measured. DOPA-positive melanocytes in the tissue were stained as previously described. 22 The tissue was washed with PBS and incubated in PBS containing 0.1% L-DOPA at 37 °C (Sigma–Aldrich Chemical, St. Louis, MO). Then, the specimens were washed in PBS for microscopic examination.

To visualize tumor-associated factors using immunohistochemical analysis, tissue specimens were incubated with factor-specific antibodies: Rabbit monoclonal anti-S100 calcium-binding protein B (1:100; GTX638273, GeneTex, Irvine, CA), goat polyclonal anti-Ki-67 antigen (1:100; sc-7846, Santa Cruz Biotechnology, Santa Cruz, CA), rabbit monoclonal anti-periostin (1:100; ab215199, Abcam, Cambridge, United Kingdom), rat monoclonal anti-vascular endothelial growth factor-A (VEGF-A, 1:100; 512902, BioLegend, San Diego, CA), mouse monoclonal anti-platelet-derived growth factor-B (PDGF-B, 1:100; sc-365805, Santa Cruz Biotechnology), goat polyclonal anti-TM (1:100; AF3894, R&D systems, Minneapolis, MN), rabbit polyclonal anti-CX3C motif chemokine receptor 1 (CX3CR1, 1:100; bs-1728R, Bioss Antibodies, Woburn, MA), rabbit monoclonal anti-VEGF receptor 1 (VEGFR1, 1:100; ab32152, Abcam), rabbit polyclonal anti-E-selectin (1:100; bs-1273R, Bioss Antibodies), rabbit polyclonal anti-L-selectin (1:100; bs-1036R, Bioss Antibodies), mouse monoclonal anti-Roundabout 4 (Robo4, 1:100; sc-166872, Santa Cruz Biotechnology), rabbit monoclonal anti-claudin 5 (1:100; ab131259, Abcam), rabbit monoclonal anti-E-cadherin (1:100; #3195, Cell Signaling Technology, Denver, MA), rabbit monoclonal anti-wingless mouse mammary tumor virus (MMTV) integration site family, member 3a (Wnt3a, 1:100; #2721, Cell Signaling Technology), rabbit monoclonal anti-β-catenin (1:100; #8480, Cell Signaling Technology), rabbit monoclonal anti-zinc finger-enhancer binding protein 1 (ZEB1, 1:100; #70512, Cell Signaling Technology), rabbit monoclonal anti-C–C chemokine receptor 7 (CCR7, 1:100; ab32527, Abcam), or rabbit monoclonal anti cluster of differentiation 163 (CD163, 1:100; ab182422, Abcam) primary antibodies. Sections were then coated with appropriate secondary antibodies (1:30 dilution, fluorescein isothiocyanate-conjugated anti-rabbit, anti-mouse, anti-rat, or anti-goat secondary antibody [Dako Cytomation, Glostrup, Denmark]) and incubated for 2 hours in the dark. Gene expressions were calculated from five random fields of constant area using the ImageJ software version 2.1.53 (NIH, Bethesda, MD). Original files were converted to monochrome 8-bit files. An arbitrary intensity threshold was set, and areas exceeding the threshold (referred to as “intensity”) were measured for each sample.

### Measurement of Metastasis-Associated Factors in the Lungs

Lung samples were isolated and homogenized in a lysis buffer (Kurabo, Osaka, Japan). The tissue extracts were centrifuged at 10,000 × *g* (Tomy MX-201; Tomy Digital Biology, Tokyo, Japan), and the supernatants were collected and analyzed. The levels of interleukin 6 (IL-6), protease-activated receptor (PAR) 1 (PAR1), PAR2, transforming growth factor β1 (TGFβ1), TGFβ receptor type I (TGFβR I), small mothers against decapentaplegic (SMAD) family member 2 (SMAD2), SMAD3, SMAD4, matrix metalloproteinase (MMP)-2 (MMP-2), MMP-9, angiopoietin-2, basic fibroblast growth factor (bFGF), vimentin, fibronectin, snail family transcriptional repressor 1 (Snail-1), inducible nitric oxide synthase (iNOS), and arginase-1 in the lung were determined using commercially available enzyme-linked immunosorbent assay kits per manufacturers' instructions: IL-6 (M6000B, R&D Systems) and angiopoietin-2 (MANG20, R&D Systems), PAR1 (MBS753326, MyBioSoutce, San Diego, CA), PAR2 (MBS4501658; MyBioSource), TGFβ1 (E-EL-M0051, Elabscience, Houston, TX), TGFβR I (Q64729, RayBiotech Life, Peachtree Corners, GA), SMAD2 (OKEH03472, Aviva Systems Biology, San Diego, CA), SMAD3 (OKEH03473, Aviva Systems Biology), SMAD4 (OKEH03425, Aviva Systems Biology), fibronectin (OKCD05702, Aviva Systems Biology), MMP-2 (ab254516, Abcam), MMP-9 (ab253227, Abcam), bFGF (bs-0217R, Bioss Antibodies), vimentin (ELK3731, ELK Biotechnology, Denver, CO), Snail-1 (LS-F2317-1, LS Bio, Shirley, MA), iNOS (CSB-E08326m, Wuhan Fine Biotech, Hubei, China), and arginase-1 (MBS2882661, MyBioSource). Optical density was measured using a microplate reader (Molecular Devices, Sunnyvale, CA).

### Measurement of Melanin Content and Western Blotting Analysis of IL-6 and Transforming Growth Factor β1 Production in In Vitro Cultured B16 Cells

To clarify the effects of FXa, EDX, and a TGFβR I (activin receptor-like kinase 5 [ALK5]) inhibitor (ALK5-I) 23 on in vitro cultured B16 cells, the amount of melanin pigments per cell was measured 48 hours after culture, and the amounts of IL-6 and TGFβ1 produced by B16 cells were analyzed using Western blotting.

For melanin measurement, 24 B16 cells were seeded in 100-mm dishes at a density of 10 ^6^ cells/dish. After overnight culture, the medium was replaced, and FXa (Sigma–Aldrich) was added at 1 U/mL to all culture dishes except for five controls. FXa-treated dishes were divided into four groups (five dishes per group): FXa only, FXa + EDX (10 mM), FXa + ALK5-I (0.3 mM: Selleck Chemicals, Houston, TX), and FXa + EDX + ALK5-I, and cultured for 72 hours. Then, B16 cells were solubilized with 1 N NaOH, and the amount of melanin in the cells was quantified based on absorbance at 400 nm and expressed as the amount of melanin per cell. Under these experimental conditions, cell viability was higher than 98% in all groups.

To measure the amounts of IL-6 and TGFβ1 produced by B16 cells cultured under the above experimental conditions by Western blotting, cultured cells were lysed by adding 50 mM Tris-HCl (pH 7.5; Sigma–Aldrich), 1.15 M NaCl (Fujifilm Wako Pure Chemical), 1% Triton X-100 (Nakarai Tesque, Kyoto, Japan), 100 μM Na _3_ VO _4_ (Nakarai Tesque), and a protease inhibitor cocktail (Nakarai Tesque) and left on ice for 10 minutes. After centrifugation at 1,500 rpm at 4 °C for 10 minutes, the resulting supernatant was used as a cell extract. Ten micrograms of total protein was collected from each cell extract and subjected to standard SDS-polyacrylamide gel electrophoresis. β-actin was added as a loading control. Plus2 Pre-Stained Protein Standard (Life Technologies, Carlsbad, CA) was also added as a protein marker. Western blotting was performed according to a previously reported method. 25 Membranes were incubated with primary antibodies against IL-6 (1:1,000; Abcam), TGFβ1 (1:1,000; Abcam), and β-actin (1:5,000; Sigma–Aldrich) for 1 hour at room temperature. After primary antibody incubation, membranes were washed and incubated with horseradish peroxidase-conjugated secondary antibodies (Novex, Frederic, MD). Immune complexes were detected using ImmunoStar Zeta reagent (Fujifilm Wako Pure Chemical), and images were acquired using the Multi Gage software version 3.0 (Fujifilm, Greenwood, GC).

### Statistical Analysis

All data are presented as the mean ± standard deviation (SD). Microsoft Excel 2010 (Microsoft Corp., Redmond, WA) was used to analyze statistical significance, along with one-way analysis of variance, followed by Tukey's post hoc test, using SPSS version 20 (SPSS Inc., Chicago, IL). Results were considered statistically significant at α = 0.05 ( *p* < 0.05) or α = 0.01 ( *p* < 0.01).

### Ethical Statement for Animal Studies

Experiments with mice were conducted in strict accordance with the recommendations of the Suzuka University of Medical Science Animal Experiment Ethics Committee (approval number 63), which were prepared according to the guidelines of the Ministry of Education, Culture, Sports, Science, and Technology of Japan. Surgery was performed under pentobarbital anesthesia, and every effort was made to minimize pain.

## Results

### Effects of Orally Administered Direct Oral Anticoagulants on B16 Cell Metastasis and Pathological Changes in Lungs

#### Effects of Direct Oral Anticoagulants on B16 Cell Metastasis Pathology

**Fig. 2:**
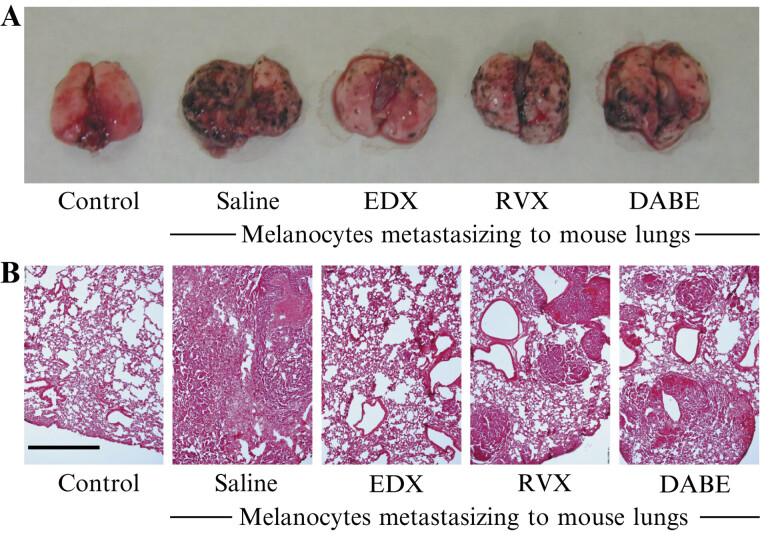
Pathological analysis of the effects of orally administered DOACs on lung metastasis in B16 cell-implanted mice. ( **A** ) Photographs of the distributions of B16 cell clusters metastasized to the lungs of mice in control, saline-, EDX-, RVX-, and DABE-treated groups. ( **B** ) Hematoxylin–eosin (HE) stained lung sections of mice in control, saline-, EDX-, RVX-, and DABE-treated groups. The length of the bar in the control of the HE-stained image indicates 100 μm. DABE, dabigatran etexilate; DOAC, direct oral anticoagulant; EDX, edoxaban; RVX, rivaroxaban.

According to experimental design 1 ( Fig. 1 ), the effects of 14-day oral administration of three DOACs to mice implanted with B16 cells on B16 cell metastasis to organs, including the lungs, were analyzed on day 15. Figure 2A shows a photograph of the distribution of B16 cell clusters metastasized to the lungs, and Fig. 2B shows an HE-stained image of the lung section. In the lungs of the control group, no black B16 cell clusters were observed, and the alveoli were clearly visible in the HE-stained images. However, numerous black clusters were observed in the lungs of the saline group; moreover, the HE-stained images revealed the absence of alveolar tissues, replaced by proliferating melanoma cells. In DOAC-treated mice, the RVX and DABE groups had fewer black clusters in the lungs than the saline group, and HE staining showed melanoma cell metastasis, with some alveolar tissue preserved. In contrast, the EDX group had fewer black clusters in the lungs, and HE staining images showed mostly intact alveolar tissue, similar to the control group.

#### Effects of Direct Oral Anticoagulants on 3,4-Dihydroxyphenylalanine-Positive Cell Distribution in the Lungs of B16 Cell-Implanted Mice

**Fig. 3:**
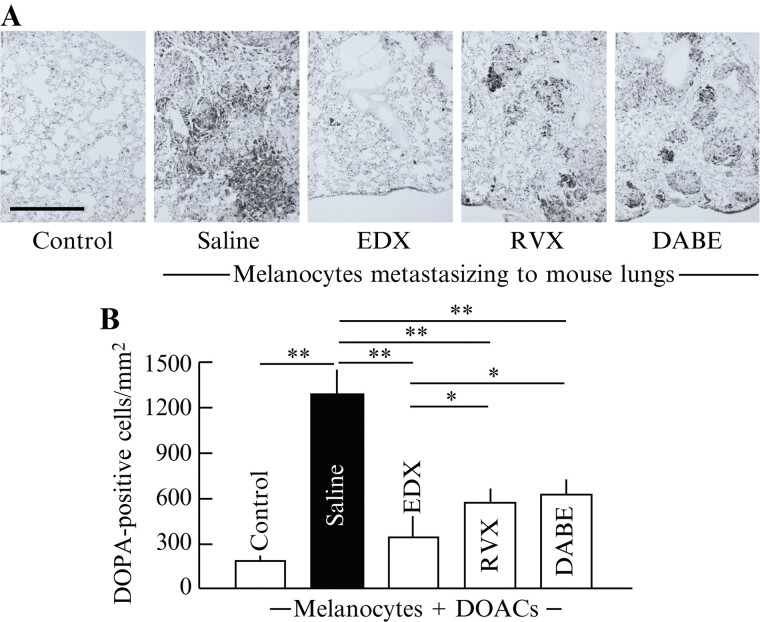
Effect of orally administered DOACs on lung metastasis in B16 cell-implanted mice. ( **A** ) DOPA-positive cell distribution in the lungs of mice in control, saline-, EDX-, RVX-, and DABE-treated groups. ( **B** ) Number of DOPA-positive cells per square millimeter in the lungs of mice in control, saline-, EDX-, RVX-, and DABE-treated groups. Data are shown as the mean ± SD. Significant differences in the number of DOPA-positive cells per square millimeter between groups are shown as * *p* < 0.05 and ** *p* < 0.01. DOAC, direct oral anticoagulant; DOPA, 3,4-dihydroxyphenylalanine; EDX, edoxaban; RVX, rivaroxaban; DABE, dabigatran etexilate; SD, standard deviation.

To quantify B16 cells in the lungs, staining images of DOPA-positive cells ( Fig. 3A ) and the density of DOPA-positive cells per square millimeter ( Fig. 3B ) in lung tissue sections were analyzed. The control group had few melanocyte clusters, with an average of <200 DOPA-positive cells per square millimeter. In contrast, the saline group had numerous DOPA-positive cells and cell clusters in the lungs, averaging >1,250 cells/mm ^2^ . Among DOAC-treated mice, both the RVX and DABE groups showed significantly fewer DOPA-positive cells than the saline group ( *p* < 0.01), averaging approximately 600 cells/mm ^2^ . The EDX-treated group showed no large number of DOPA-positive cell clusters, with an average of <400 cells/mm ^2^ . This reduction was highly significant compared with the number of cells in the saline group ( *p* < 0.01) and significant compared with that in the RVX and DABE groups ( *p* < 0.05).

#### Effects of Direct Oral Anticoagulants on S100B-Stained Cell Distribution in the Lungs of B16 Cell-Implanted Mice

**Fig. 4:**
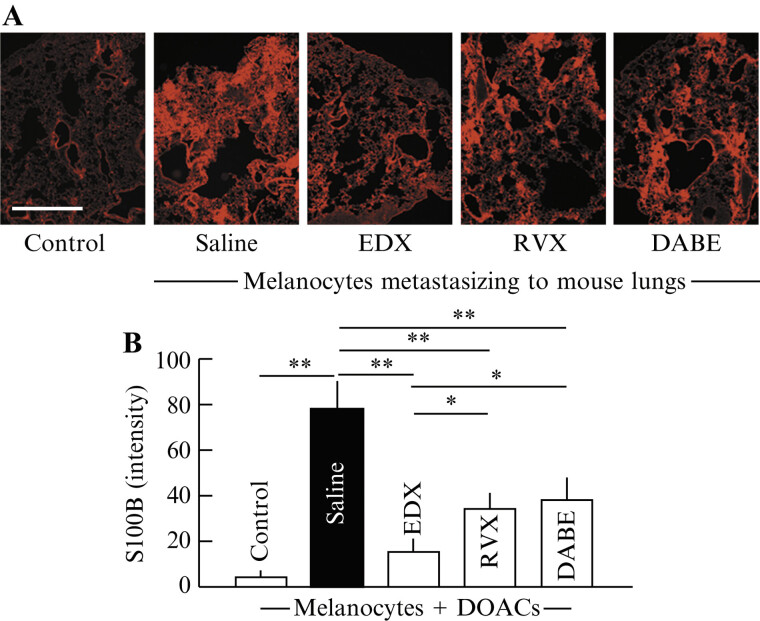
Immunohistochemical analysis of the effects of orally administered DOACs on lung metastasis in B16 cell-implanted mice. ( **A** ) Immunohistochemical analysis of melanoma-specific marker (S100B-stained cells) in the lungs of mice in control, saline-, EDX-, RVX-, and DABE-treated groups. ( **B** ) S100B-stained cell intensity, obtained from data shown in ( **A** ), in the lungs of mice in control, saline-, EDX-, RVX-, and DABE-treated groups. Data are shown as the mean ± SD. Significant differences in number of DOPA-positive cells per square millimeter between groups are shown as * *p* < 0.05 and ** *p* < 0.01. DABE, dabigatran etexilate; DOAC, direct oral anticoagulant; DOPA, 3,4-dihydroxyphenylalanine; EDX, edoxaban; RVX, rivaroxaban; SD, standard deviation.

To further quantify metastatic B16 cells in the lungs, we analyzed the staining of S100B-positive cells, a marker highly expressed in malignant melanocytes, in lung tissue sections ( Fig. 4A ) and the density of S100B-positive cells per square millimeter ( Fig. 4B ). In control mice, S100B staining and relative intensity were <5, whereas saline group mice showed a significantly higher staining intensity, with mean relative intensities >75. In contrast, DOAC-treated mice had mean staining intensities of approximately 35 to 40 in the RVX and DABE groups, which were highly significantly ( *p* < 0.01) decreased compared with those in the saline group. The EDX group had mean staining intensities of approximately 17, which was highly significantly decreased compared with that in the saline group ( *p* < 0.01) and significantly decreased compared with that in the RVX and DABE groups ( *p* < 0.05).

#### Effects of Direct Oral Anticoagulants on Ki67-Stained Cell Distribution in the Lungs of B16 Cell-Implanted Mice

**Fig. 5:**
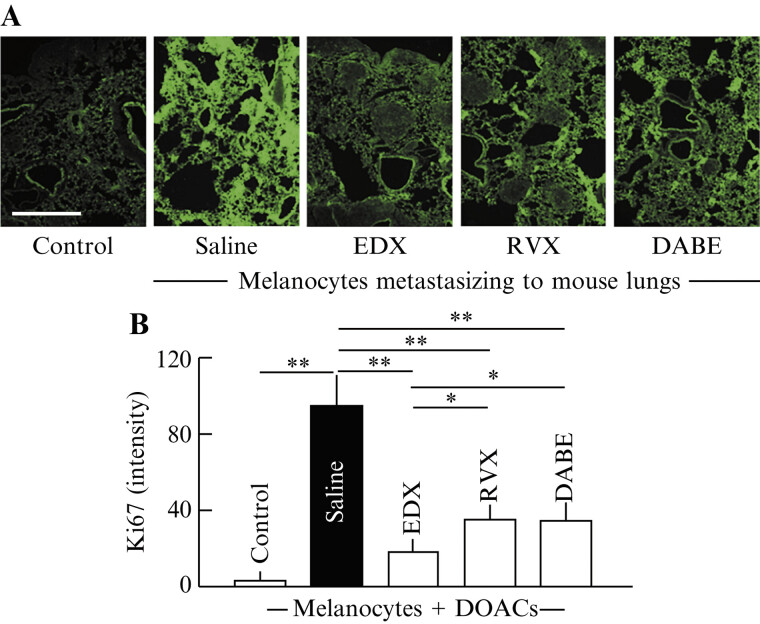
Immunohistochemical analysis of the effects of orally administered DOACs on lung metastasis in B16 cell-implanted mice. ( **A** ) Immunofluorescence data of a proliferation ability marker of melanoma cells (Ki67-stained cells) in the lungs of mice in control, saline-, EDX-, RVX-, and DABE-treated groups. ( **B** ) Ki67-stained cell intensity, obtained from data shown in ( **A** ), in the lungs of mice in control, saline-, EDX-, RVX-, and DABE-treated groups. Data are shown as the mean ± SD. Significant differences in the number of DOPA-positive cells per square millimeter between groups are shown as * *p* < 0.05 and ** *p* < 0.01. DABE, dabigatran etexilate; DOAC, direct oral anticoagulant; DOPA: 3,4-dihydroxyphenylalanine; EDX, edoxaban; RVX, rivaroxaban; DABE, dabigatran etexilate; SD, standard deviation.

To quantify the proliferation potential of B16 cells in the lungs, we analyzed the staining pattern of Ki67-positive cells, a tumor proliferation ability marker ( Fig. 5A ), and the relative Ki67 staining intensity ( Fig. 5B ) in lung tissue sections. Control mice had relative staining intensities <5, whereas the saline group exhibited high staining with mean relative intensities >95. Among DOAC-treated mice, the staining intensity in the RVX and DABE groups decreased to an average of 35 to 37, which was highly significantly different from that in the saline group ( *p* < 0.01). Furthermore, in the EDX group mice, the mean staining intensity in lung tissue decreased to an average of 18, which was highly significantly different from that in the saline group ( *p* < 0.01) and significantly different from those in the RVX and DABE groups ( *p* < 0.05).

#### Effects of Direct Oral Anticoagulants on Plasma Levels of Inflammatory Factors in B16 Cell-Implanted Mice

**Fig. 6:**
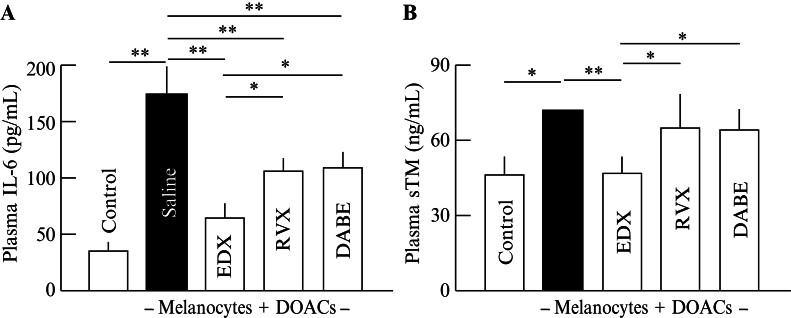
Effects of orally administered DOACs on IL-6 and sTM plasma concentrations in B16 cell-implanted mice. ( **A** ) IL-6 plasma concentrations (pg/mL) of mice in control, saline-, EDX-, RVX-, and DABE-treated groups. ( **B** ) sTM plasma concentrations (ng/mL) of mice in control, saline-, EDX-, RVX-, and DABE-treated groups. Data are shown as the mean ± SD. Significant differences in plasma IL-6 and sTM concentrations between groups are shown as * *p* < 0.05 and ** *p* < 0.01. DABE, dabigatran etexilate; DOAC, direct oral anticoagulant; DOPA, 3,4-dihydroxyphenylalanine; EDX, edoxaban; IL-6, interleukin 6; RVX, rivaroxaban; SD, standard deviation; sTM, soluble thrombomodulin.

To examine the effects of DOAC on in vivo inflammation in B16 cell-inoculated mice, plasma concentrations of the inflammatory marker IL-6 ( Fig. 6A ) and soluble thrombomodulin (sTM; Fig. 6B ), which indicate the degree of inflammatory damage in the vascular endothelium, were measured. The plasma IL-6 level averaged 36 pg/mL in control mice, and significantly ( *p* < 0.01) increased to 175 pg/mL on average in the saline group mice. In DOAC-treated mice, IL-6 levels significantly ( *p* < 0.01) decreased to approximately 110 pg/mL on average in the DVX and DABE groups compared with the saline group. Furthermore, IL-6 levels decreased to approximately 70 pg/mL on average in the EDX group, which was highly significantly lower than that in the saline group ( *p* < 0.01) and significantly lower than those in the RVX and DABE groups ( *p* < 0.05).

Plasma sTM levels averaged 45 pg/mL in the control group and increased to 73 pg/mL in the saline group. In DOAC-treated mice, the sTM levels in the RVX and DABE groups were reduced to approximately 65 pg/mL on average, a significant decrease compared with the levels in the saline group ( *p* < 0.05). In contrast, the sTM levels in the EDX group decreased to the same level as those in the control group, a significant decrease compared with the levels in the saline ( *p* < 0.01) and RVX and DABE groups ( *p* < 0.05). These data indicate that DOAC treatment, particularly EDX, significantly suppressed inflammation in tumor-bearing mice.

These results showed that DOAC treatment suppressed melanoma cell metastasis in mice. We subsequently analyzed the mechanism by which EDX, which had the strongest metastasis suppression effect, inhibits tumor metastasis.

### Effect of Orally Administered Edoxaban on Organ Metastasis of B16 Cells and Analysis of the Inhibitory Mechanism of Edoxaban

#### Effect of Edoxaban on B16 Cell Metastasis Pathology

**Fig. 7:**
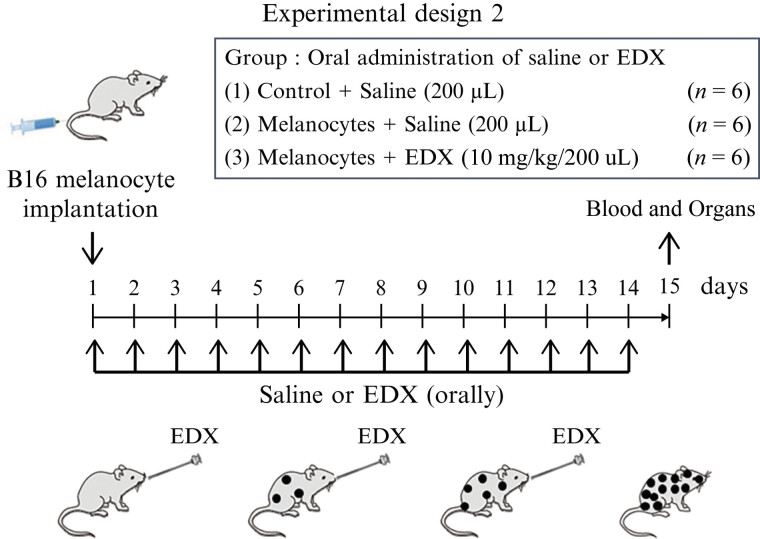
Experimental design 2 evaluates the effects of EDX on lung metastasis in B16 cell-implanted female C57BL/6j mice. Cells (100 μL, 1 × 10 ^6^ ) were slowly injected into mice tail veins. Simultaneously, five mice were orally administered 200 μL of saline for 14 days (saline group) or EDX (10 mg/kg bw) for 14 days (EDX group). Five untreated mice served as the control group. On the 15th day, each mouse was anesthetized and underwent surgery to collect heparinized blood and organs, including lungs, liver, colon, and ovaries. bw, body weight; EDX, edoxaban.

We conducted an experiment as shown in Fig. 7 . An untreated control group ( *n* = 6), saline-treated mice implanted with B16 cells (saline group; *n* = 6), and mice treated with EDX (200 μL, 10 mg/kg bw/day) for 14 days (EDX group; *n* = 6) were evaluated. On the 15th day, the mice underwent abdominal incision under anesthesia, blood and organs were collected, and heparinized plasma and organs were stored at −80 °C for later use.

**Fig. 8:**
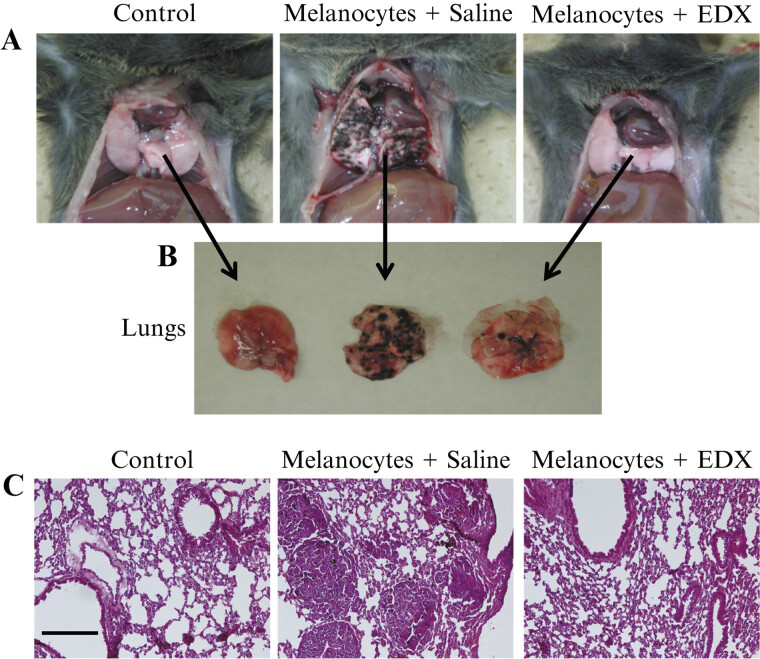
Pathological analysis of the effects of orally administered EDX on lung metastasis in B16 cell-implanted mice. ( **A** ) Photographs of B16 cell clusters in the lungs of mice in control, saline, and EDX groups. ( **B** ) Photographs of B16 cell clusters in isolated lungs of mice in control, saline, and EDX groups. ( **C** ) Hematoxylin–eosin (HE) stained image of the lung section of mice in the control, saline, and EDX groups. The length of the bar in the control of the HE-stained image indicates 100 μm. EDX, edoxaban.

As shown in Fig. 8A, B , the high number of B16 cell clusters observed in the lungs of saline group mice significantly decreased in the lungs of EDX group mice. Figure 8C shows HE-stained lung sections, where the saline group exhibited extensive B16 cell clusters and reduced alveolar tissue, whereas the EDX group generally maintained alveolar tissue.

**Fig. 9:**
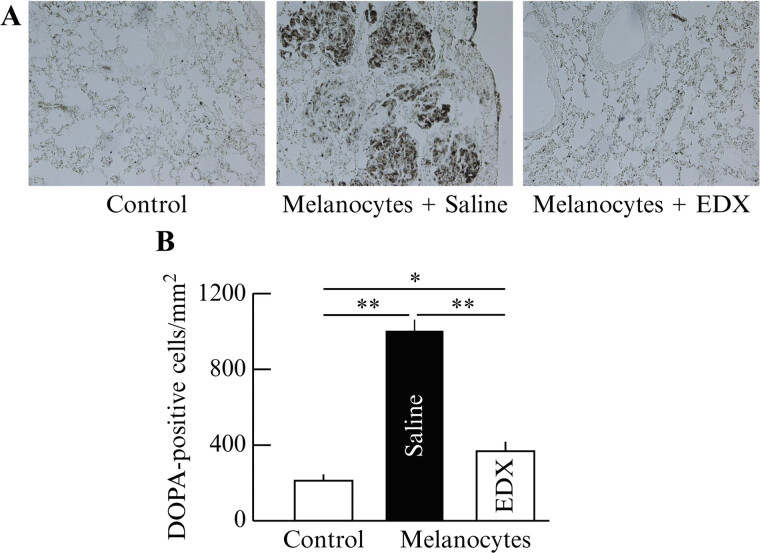
Effect of orally administered EDX on lung metastasis in B16 cell-implanted mice. ( **A** ) DOPA-positive cell distribution in the lungs of mice in control, saline, and EDX groups. ( **B** ) Number of DOPA-positive cells per square millimeter in the lungs of mice in control, saline, and EDX groups. Data are shown as the mean ± SD. Significant differences in the number of DOPA-positive cells per square millimeter between groups are shown as * *p* < 0.05 and ** *p* < 0.01. DOPA, 3,4-dihydroxyphenylalanine; EDX, edoxaban.

Staining images of DOPA-positive cells ( Fig. 9A ) indicated that the saline group exhibited numerous large, clearly visible DOPA-positive cell clusters with destroyed lung tissue, whereas the EDX group had largely preserved lung tissue similar to the control group, with no large DOPA-positive cell clusters and only a few positive cells. The number of DOPA-positive cells per square millimeter ( Fig. 9B ) indicated that the control group had an average of approximately 200 DOPA-positive cells per square millimeter of lung tissue, which significantly ( *p* < 0.01) increased to approximately 1,030 cells/mm ^2^ in the saline group. In contrast, those in the EDX group significantly ( *p* < 0.01) decreased to approximately 380 cells/mm ^2^ .

To clarify the mechanism by which EDX suppresses B16 cell metastasis, we first measured plasma and intracellular signaling factors believed to be involved in B16 cell metastasis via the major FXa receptor PAR2. Next, we measured angiogenesis-related factors from tumor cells, surrounding tissue cells, and vascular endothelial cells involved in metastasis. Furthermore, we measured epithelial–mesenchymal transition (EMT)-related factors 26 and tumor-associated macrophages (TAMs)-related factors, 27 which are deeply involved in metastasis.

#### Effects of Edoxaban on the Expression of Plasma and Intracellular Tumor-Associated Signaling Factors in Lung Tissues of B16 Cell-Implanted Mice

PAR2 has been reported to induce cancer progression in both TGFβ signaling-dependent and independent manners, 28 and to regulate the expression of TGFβR I (= ALK5). 29 Therefore, we first investigated the effect of EDX treatment on PAR1, PAR2, and TGFβ-related factor expression, which are believed to be involved in B16 cell metastasis, in mice plasma or lung tissue.

Table 1 shows the mean ± SD results of plasma and lung factor levels, presumed to be related to B16 cell metastasis via the PAR2 pathway, in the control, saline-treated, and EDX-treated groups. Following B16 cell implantation, plasma IL-6 and TGFβ1 levels, along with lung tissue levels of PAR1, PAR2, TGFβ1, TGFβR I, SMAD2, SMAD3, and SMAD4, all significantly ( *p* < 0.01) increased in the saline group compared with the control group. In contrast, all plasma and lung factors except PAR1 significantly ( *p* < 0.05 or 0.01) decreased in the EDX group compared with the saline group.

**Table 1:** Effect of edoxaban on the expression of tumor-associated signaling factors in the plasma and lungs of mice implanted with B16 melanoma cells

	Control	Melanocytes + saline	Melanocytes + EDX
Plasma IL-6 (pg/mL)	12.4 ± 2.4	123.4 ± 29.8 a	36.2 ± 13.8 b
Plasma TGFβ1 (pg/mL)	12.2 ± 2.4	50.3 ± 6.7 a	37.7 ± 3.7 a d
Lung PAR1 (ng/mg protein)	13.2 ± 3.0	85.9 ± 9.6 a	67.0 ± 5.2 a
Lung PAR2 (ng/mg protein)	20.4 ± 7.0	129.6 ± 19.3 a	23.5 ± 9.4 a b
Lung TGFβ1 (pg/mg protein)	5.4 ± 0.7	18.6 ± 2.1 a	7.1 ± 1.7 b
Lung TGFβR I (pg/mg protein)	16.2 ± 5.1	73.0 ± 17.0 a	39.7 ± 8.2 c d
Lung SMAD2 (ng/mg protein)	33.4 ± 9.1	81.5 ± 8.7 a	33.4 ± 9.1 b c
Lung SMAD3 (ng/mg protein)	28.2 ± 7.3	56.5 ± 5.4 a	42.8 ± 5.5 b d
Lung SMAD4 (ng/mg protein)	31.7 ± 8.1	77.3 ± 7.7 a	55.5 ± 7.5 b c

Abbreviations: EDX, edoxaban; IL-6, interleukin 6; PAR, protease-activated receptor; SMAD, small mothers against decapentaplegic; TGFβ1, transforming growth factor β1; TGFβR I, TGFβ receptor type I.

Each value represents the mean ± standard deviation.

Values shown as ^a^ *p* < 0.01 and ^c^ *p* < 0.05 indicate significant differences against the control based on Tukey's test.

Values shown as ^b^ *p* < 0.01 and ^d^ *p* < 0.05 indicate significant differences against the melanocytes + saline group based on Tukey's test.

#### Effects of Edoxaban on Plasma and Lung Tissue Levels of Angiogenic Factors Expressed by Tumor and Surrounding Cells in B16 Cell-Implanted Mice

Tumor cell metastasis is accompanied by increased angiogenesis in the tumor and surrounding cells. We next measured MMP-2 and MMP-9 plasma levels, along with lung tissue levels of periostin (a protein composing the extracellular matrix that supports epithelial cell adhesion and migration), angiopoietin-2 (a membrane protein that acts together with VEGF to induce the migration and proliferation of vascular endothelial cells and angiogenesis), L-selectin (a cell adhesion molecule widely expressed on leukocytes and regulates their adhesion to endothelial cells, migration, and signal transduction), VEGF-A (a glycoprotein secreted by tumor and other cells playing an important role in angiogenesis and vasculogenesis), bFGF (a fibroblast growth factor involved in angiogenesis and wound healing), and PDGF-B (a protein regulating mesenchymal cell proliferation and migration that forms a dimeric isoform together with PDGF-A and binds to platelet-derived growth factor receptor (PDGFR) to transmit signals, which are believed to be associated with tumor angiogenesis and are mainly derived from tumor and surrounding cells). As shown in Table 2 , saline group mice showed a significant increase in blood and tissue levels of all measured factors compared with control group mice ( *p* < 0.01). In contrast, EDX group mice showed a significant decrease in all factors in plasma and lung tissue compared with saline group mice ( *p* < 0.05 or 0.01).

**Table 2:** Effect of edoxaban on the expression of tumor angiogenesis-associated factors in tumor cells and surrounding cells of mice implanted with B16 melanoma cells

	Control	Melanocytes + saline	Melanocytes + EDX
Plasma MMP-2 (pg/mL)	108.8 ± 20.4	340.0 ± 44.2 a	154.6 ± 35.9 b
Plasma MMP-9 (pg/mL)	99.2 ± 16.1	176.8 ± 20.2 a	93.9 ± 8.6 b
Lung periostin (intensity)	4.0 ± 2.1	75.7 ± 14.6 a	20.2 ± 7.5 b c
Lung angiopoetin-2 (ng/mg protein)	8.5 ± 1.0	31.2 ± 7.3 a	14.9 ± 3.7 b
Lung L-selectin (intensity)	52.2 ± 6.1	265.6 ± 44.7 a	76.5 ± 8.6 b
Lung VEGFA (intensity)	41.5 ± 10.1	224.4 ± 44.0 a	125.2 ± 16.8 c d
Lung bFGF (pg/mg protein)	6.9 ± 2.8	46.0 ± 8.8 a	15.6 ± 6.0 b
Lung PDGF-B (intensity)	6.7 ± 2.5	70.9 ± 9.8 a	9.2 ± 2.4 b

Abbreviations: bFGF, basic fibroblast growth factor; EDX, edoxaban; L-selectin, leukocyte-selectin; MMP, matrix metalloproteinases; PDGF-B, platelet-derived growth factor-B; VEGFA, vascular endothelial growth factor A.

Each value represents the mean ± standard deviation.

Values shown as ^a^ *p* < 0.01 and ^c^ *p* < 0.05 indicate significant differences against the control based on Tukey's test.

Values shown as ^b^ *p* < 0.01 and ^d^ *p* < 0.05 indicate significant differences against the melanocytes + saline group based on Tukey's test.

#### Effects of Edoxaban on the Expression of Factors Related to Angiogenesis and Tumor Cell Invasion in Endothelial Cells of Lung Tissues of B16 Cell-Implanted Mice

Several factors of vascular endothelial cells play important roles in tumor metastasis and tissue invasion. We next analyzed the effect of EDX on the expression of intracellular adhesion molecules, angiogenesis factor receptors, and intercellular junction molecules expressed in vascular endothelial cells. As shown in Table 3 , saline group mice showed significant ( *p* < 0.05 or 0.01) increases in the levels of TM (a membrane protein of endothelial cells involved in regulation of blood coagulation, fibrinolysis, inflammation, and angiogenesis), CX3CR1 (a chemokine receptor that promotes migration of cells such as leukocytes), VEGFR1 (a VEGF receptor expressed in vascular endothelial cells and inflammatory cells, which is involved in pathological angiogenesis), E-selectin (a cell adhesion molecule expressed on cytokine-activated vascular endothelial cells), and Robo4 (a transmembrane molecule present in vascular endothelial cells and involved in cell migration, proliferation, angiogenesis, and vascular permeability), which are believed to be expressed in lung vascular endothelial cells, compared with control mice. In contrast, EDX-treated mice showed significant ( *p* < 0.05 or 0.01) decreases in these factors. Further, the levels of the intercellular tight junction molecule claudin 5 (a transmembrane molecule present in the tight junctions of vascular endothelial cells) and E-cadherin (a cell membrane molecule involved in tight junctions between cells that regulates epithelial cell proliferation, differentiation, and survival) significantly ( *p* < 0.05 or 0.01) decreased in the lungs of saline group mice compared with control mice but significantly ( *p* < 0.01) increased in EDX-treated mice compared with saline-treated mice.

**Table 3:** Effect of edoxaban on the expression of tumor angiogenesis/invasion-associated factors in pulmonary vascular endothelial cells of mice implanted with B16 melanoma cells

	Control	Melanocytes + saline	Melanocytes + EDX
Lung TM (intensity)	112.9 ± 8.6	306.9 ± 25.7 a	194.1 ± 13.2 b c
Lung CX3CR1 (intensity)	5.5 ± 0.5	52.9 ± 4.8 b	21.7 ± 1.5 a d
Lung VEGFR1 (intensity)	5.3 ± 0.9	179.4 ± 14.8 a	52.1 ± 12.8 a c
Lung E-selectin (intensity)	5.0 ± 0.8	31.5 ± 2.4 a	14.7 ± 2.7 b c
Lung Robo4 (intensity)	2.8 ± 1.2	66.6 ± 8.0 a	11.8 ± 4.4 c
Lung claudin 5 (intensity)	32.5 ± 4.1	4.6 ± 2.2 a	25.4 ± 3.9 c
Lung E-cadherin (intensity)	16.9 ± 2.0	8.8 ± 1.3 b	24.5 ± 3.4 b c

Abbreviations: CX3CR1, chemokine CX3C receptor 1; E-cadherin, epithelial-cadherin; E-selectin, endothelial cell-selectin; EDX, edoxaban; Robo4, Roundabout 4; TM, thrombomodulin; VEGFR1, vascular endothelial growth factor receptor 1.

Each value represents the mean ± standard deviation.

Values shown as ^a^ *p* < 0.01 and ^b^ *p* < 0.05 indicate significant differences against the control based on Tukey's test.

Values shown as ^c^ *p* < 0.01 and ^d^ *p* < 0.05 indicate significant differences against the melanocytes + saline group based on Tukey's test.

#### Effects of Edoxaban on Epithelial–Mesenchymal Transition-Associated Factor Expression in the Lung Tissues of B16 Cell-Implanted Mice

As EMT events are closely related to tumor invasion and metastasis, 26 we next evaluated the effect of EDX on EMT-associated factor levels in the lung tissue of B16 cell-implanted mice.

As shown in Table 4 , saline-treated mice showed significant ( *p* < 0.01) increases in the levels of vimentin (an EMT protein involved in cell–cell communication, adhesion, and cell morphology, which functions as a mediator of the Wnt signaling pathway), fibronectin (an EMT protein that controls cell adhesion, spreading, migration, proliferation, and differentiation), Snail-1 (a protein that causes cells to lose epithelial cell characteristics and acquire mesenchymal cell characteristics during EMT), Wnt3a (an EMT protein involved in tumorigenesis, developmental processes, cell fate control, and embryonic patterning), β-catenin (a protein involved in cell–cell communication and adhesion, which plays a role in cell morphology during EMT), and ZEB1 (a protein that induces EMT by suppressing the adhesion molecule β-catenin) in the lung tissue compared with control mice. In contrast, EDX-treated mice showed significant ( *p* < 0.05 or 0.01) decreases in the levels of all of these factors compared with saline-treated mice.

**Table 4:** Effect of edoxaban on the expression of epithelial–mesenchymal transition-associated factors in lung tissues of mice implanted with B16 melanoma cells

	Control	Melanocytes + saline	Melanocytes + EDX
Lung vimentin (ng/mg protein)	11.3 ± 5.4	50.1 ± 10.5 a	15.0 ± 4.8 b
Lung fibronectin (ng/mg protein)	16.2 ± 5.0	57.2 ± 7.8 a	22.6 ± 5.1 b
Lung Snail-1 (ng/mg protein)	11.8 ± 5.0	37.7 ± 5.4 a	12.0 ± 3.0 b
Lung Wnt3a (intensity)	2.9 ± 0.4	143.1 ± 6.7 a	56.5 ± 4.1 a b
Lung β-catenin (intensity)	7.0 ± 0.7	27.8 ± 2.6 a	16.4 ± 1.6 c d
Lung ZEB1 (intensity)	1.5 ± 0.3	17.4 ± 1.7 a	4.1 ± 0.8 b c

Abbreviations: EDX, edoxaban; Snail-1, small family zinc finger 1; Wnt3a, wingless MMTV integration site, family member 3a; ZEB1, zinc finger E-box binding homeobox 1.

Each value represents the mean ± standard deviation.

Values shown as ^a^ *p* < 0.01 and ^c^ *p* < 0.05 indicate significant differences against the control based on Tukey's test.

Values shown as ^b^ *p* < 0.05 and ^d^ *p* < 0.01 indicate significant differences against the melanocytes + saline group by Tukey's test.

#### Effects of Edoxaban on Tumor-Associated Macrophage Expression in the Lung Tissues of B16 Cell-Implanted Mice

In the tumor microenvironment, various immune and stromal cells, such as fibroblasts and vascular endothelial cells, interact with tumor cells, with TAMs playing important roles in tumor growth and progression. 27 To clarify which type of TAM is involved in suppressing melanoma cell metastasis in B16 cell-transplanted mice, we analyzed changes in the lung molecular markers involved in each type.

As shown in Table 5 , the M1 macrophage-associated markers CCR7 (a chemokine receptor associated with increased frequency of lymph node metastasis) and iNOS showed no significant changes in either saline- or EDX-treated mice compared with controls. In contrast, the M2 macrophage-associated markers CD163 (a macrophage molecule related to tumor growth and malignancy) and arginase-1 (a hydrolase that converts arginine into ornithine and urea) significantly increased and decreased in saline- and EDX-treated mice, respectively. These findings indicate that M2 macrophages are involved in TAM-mediated EDX suppression of melanoma cell metastasis in B16 cell-transplanted mice.

**Table 5:** Effect of edoxaban on tumor-associated macrophages expression in the lungs of mice implanted with B16 melanoma cells

	Control	Melanocytes + saline	Melanocytes + EDX
M1 macrophages			
Lung CCR7 (intensity)	7.0 ± 2.0	11.8 ± 4.6	10.9 ± 1.8
Lung iNOS (IU/mg protein)	46.8 ± 9.9	51.8 ± 10.4	52.0 ± 7.2
M2 macrophages			
Lung CD163 (intensity)	7.1 ± 1.6	48.8 ± 7.0 a	25.5 ± 5.7 a b
Lung arginase-1 (pg/mg protein)	11.0 ± 3.7	41.0 ± 5.9 a	23.9 ± 5.48 a b

Abbreviations: CCR7, C–C chemokine receptor type 7; CD163, cluster of differentiation 163; EDX, edoxaban; iNOS, inducible nitric oxide synthase.

Each value represents the mean ± standard deviation.

Values shown as ^a^ *p* < 0.01 indicate significant differences against the control based on Tukey's test. Values shown as ^b^ *p* < 0.01 indicate significant differences against the melanocytes + saline group based on Tukey's test.

#### Effects of Edoxaban and Transforming Growth Factor β Receptor Type I on Melanoma Metastasis in the Lung Tissues of B16 Cell-Implanted Mice

PAR2 has been implicated in cancer progression through both TGFβ signal-dependent and independent ways, 28 with TGFβ itself promoting tumor formation and metastasis in advanced tumor stages. TGFβR is composed of two subunits, TGFβR type I (TGFβR I, the same as ALK5) and type II (TGFβR II). The TGFβ intracellular signal is transmitted by forming a complex between the two subunits. Inhibition of this complex formation blocks TGFβ-mediated signaling. Therefore, to clarify whether the TGFβ-mediated metastasis pathway is dependent on PAR2-driven tumor metastasis, we analyzed the effects of EDX and ALK5-I, which inhibits complex formation between TGFβR I and TGFβR II, on B16 cell metastasis. 29

**Fig. 10:**
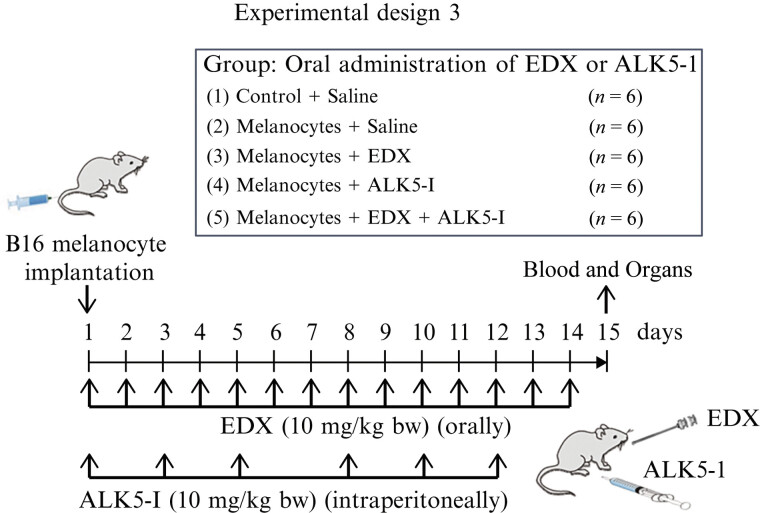
Experimental design 3 evaluates the effects of EDX and ALK5-I on lung metastasis in B16 cell-implanted female C57BL/6j mice. B16 cell-implanted mice were administered saline alone (orally), EDX (10 mg/kg mouse bw, orally), ALK5-I (10 mg/kg bw, intraperitoneally), or EDX (10 mg/kg mouse bw, orally) + ALK5-I (10 mg/kg bw, intraperitoneally) daily for 14 days. Five untreated mice served as the control group. On the 15th day, each mouse was anesthetized and underwent surgery to collect and analyze heparinized blood and lungs. bw, body weight; ALK5-I, ALK5 inhibitor; EDX, edoxaban.

**Fig. 11:**
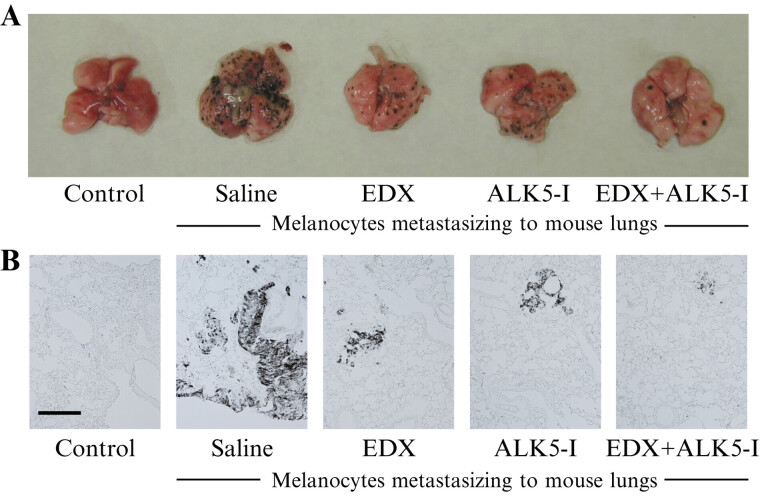
Effect of saline, EDX alone, ALK5-I alone, and EDX + ALK5-I on lung metastasis in B16 cell-implanted mice. ( **A** ) Photographs of B16 cell clusters in isolated lungs of mice in control, saline-, EDX-, ALK5-I-, and EDX + ALK5-I-treated groups. ( **B** ) DOPA-positive cell distribution in the lungs of mice in control, saline-, EDX-, ALK5-I-, and EDX + ALK5-I-treated groups. ALK5-I, ALK5 inhibitor; DOPA, 3,4-dihydroxyphenylalanine; EDX, edoxaban.

The experiment was performed as shown in Fig. 10 . Melanocyte-implanted mice were administered either saline (orally), EDX (10 mg/kg bw; orally), ALK5-I (10 mg/kg bw; intraperitoneally), or EDX (10 mg/kg bw; orally) + ALK5-I (10 mg/kg bw; intraperitoneally) for 14 days. On the 15th day, heparinized blood was collected under anesthesia, and the lungs were removed. Metastasized melanocytes were quantified via DOPA staining images and the number of DOPA-positive cells, while plasma IL-6 concentrations were measured. Figure 11 shows the effects of saline, EDX, ALK5-I, and EDX + ALK5-I on B16 cell metastasis in the lungs of mice 15 days postimplantation: Fig. 11A shows images of B16 cell metastasis, and Fig. 11B shows staining images of DOPA-positive cells in the lungs. A large number of melanocytes were observed in the lung tissue of saline-treated mice (saline group). In contrast, the number of melanocytes in the lung tissue of EDX- or ALK5-I-treated mice significantly decreased. Similarly, the number of DOPA-positive cells in the lungs of mice administered EDX + ALK5-I significantly decreased.

**Fig. 12:**
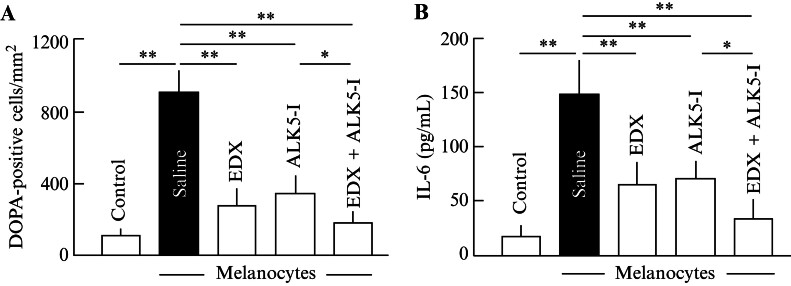
Effects of saline, EDX alone, ALK5-I alone, and EDX + ALK5-I on DOPA-positive cells in the lungs and IL-6 plasma concentrations in B16 cell-implanted mice. ( **A** ) Number of DOPA-positive cells/mm ^2^ in the lungs of mice in control, saline-, EDX-, ALK5-I-, and EDX + ALK5-I-treated groups. ( **B** ) Plasma IL-6 concentrations (pg/mL) of mice in control, saline-, EDX-, ALK5-I-, and EDX + ALK5-I-treated groups. Data are shown as the mean ± SD. Significant differences in the number of DOPA-positive cells and plasma IL-6 concentrations between groups are shown as * *p* < 0.05 and ** *p* < 0.01. ALK5-I, ALK5 inhibitor; DOPA, 3,4-dihydroxyphenylalanine; EDX, edoxaban; IL-6, interleukin 6; SD, standard deviation.

Quantitative changes in DOPA-positive cells in the lungs are shown in Fig. 12A . Approximately 900 DOPA-positive cells were observed in the lungs of mice in the saline group, whereas the cell numbers were significantly ( *p* < 0.01) reduced to approximately 250 and 320 in EDX- and ALK5-I-treated mice, respectively. The number of DOPA-positive cells in the lungs of mice administered EDX + ALK5-I was approximately 180, which was significantly smaller than that in the saline ( *p* < 0.01) and ALK5-I groups ( *p* < 0.05). As shown in Fig. 12B , the mean plasma IL-6 level in the saline group was approximately 150 μg/mL, which was significantly ( *p* <0.01) higher than that in the control group, while the mean IL-6 levels in the EDX and ALK5-I groups were approximately 70 μg/mL, both of which were significantly ( *p* <0.01) lower than that in the saline group. Furthermore, the mean IL-6 level in the EDX + ALK5-I group was approximately 30 μg/mL, which was also significantly ( *p* < 0.05) lower than that in the ALK5-I group. The number of DOPA-positive cells and IL-6 levels in the EDX + ALK5-I group were both reduced to approximately half of those in the EDX and ALK5-I groups, indicating that EDX and ALK5-I additively suppress melanoma cell metastasis and inflammation.

#### Effects of Factor Xa, Edoxaban, and ALK5-I on Melanin Content, and Interleukin 6 and Transforming Growth Factor β1 Production in B16 Cells

**Fig. 13:**
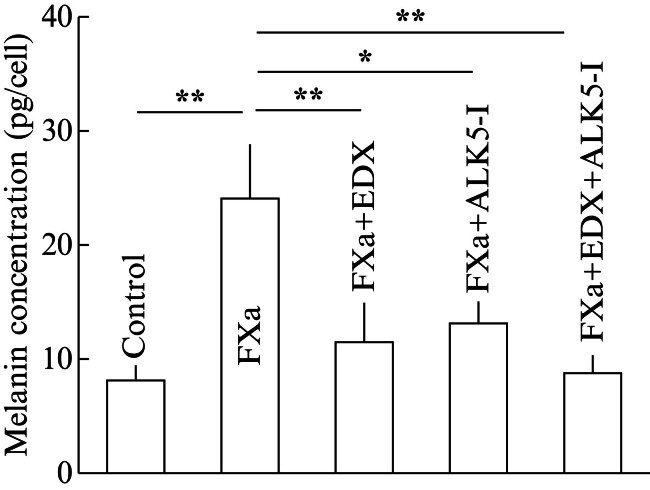
Effect of treatment with FXa, FXa + EDX, FXa + ALK5-I, or FXa + EDX + ALK5-I on melanin production in in vitro cultured B16 cells. The control group was not treated with FXa. Data are shown as the mean ± SD. Significant differences in melanin concentration (pg/cell) between groups are shown as * *p* < 0.05 and ** *p* < 0.01. ALK5-I, ALK5 inhibitor; EDX, edoxaban; FXa, factor Xa; SD, standard deviation.

Figure 13 shows the effects of FXa, FXa + EDX, FXa + ALK5-I, and FXa + EDX + ALK5-I on melanin production in B16 cells. The melanin concentration per melanoma cell was significantly ( *p* < 0.01) increased in the FXa treatment group compared with the control group, whereas it was significantly ( *p* < 0.01) decreased by approximately 45% in the FXa + EDX treatment group, by approximately 50% in the FXa + ALK5-I treatment group, and to the same level as in the control group in the FXa + EDX + ALK5-I treatment group. These results suggest that EDX and ALK5-I additively inhibit FXa-dependent melanin production in B16 cells.

**Fig. 14:**
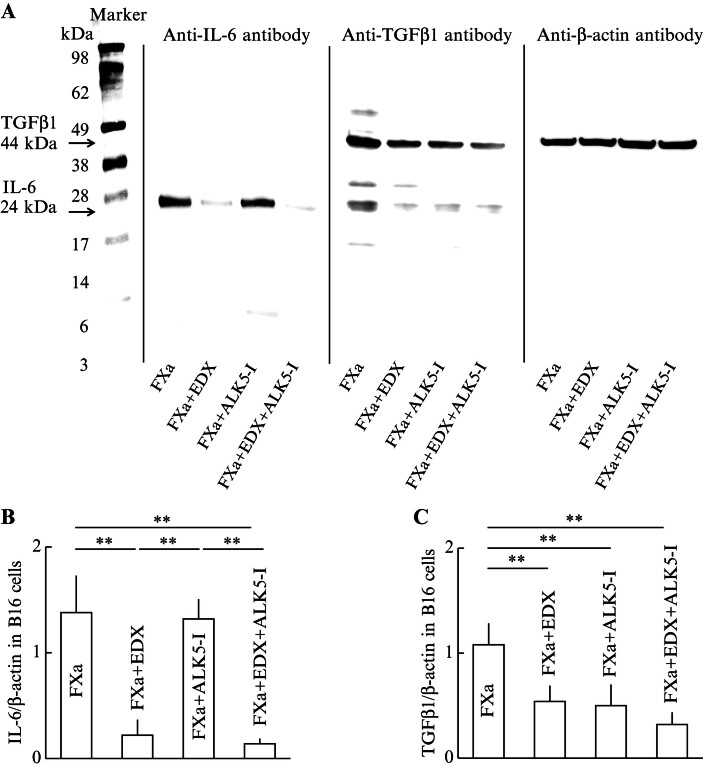
( **A** ) Western blotting analysis of the effects of FXa, FXa + EDX, FXa + ALK5-I, and FXa + EDX + ALK5-I on IL-6, TGFβ1, and β-actin production in B16 cells. After protein transfer, the membranes were treated with primary antibodies against IL-6, TGFβ1, or β-actin, and then with horseradish peroxidase-conjugated secondary antibodies. Immune complexes were detected as described in the text. ( **B** ) IL-6/β-actin ratio and ( **C** ) TGFβ1/β-actin ratio in each group. These ratios were determined by identifying IL-6, TGFβ1, and β-actin on Western blotting membranes from proteins produced in B16 cells cultured in the presence of FXa, FXa + EDX, FXa + ALK5-I, or FXa + EDX + ALK5-I, and measuring their band densities. Data are shown as the mean ± SD. Significant differences in IL-6/β-actin ratio or TGFβ1/β-actin ratio between groups are shown as ** *p* < 0.01. ALK5-I, ALK5 inhibitor; EDX, edoxaban; FXa, factor Xa; IL-6, interleukin 6; TGFβ1, transforming growth factor β1; SD, standard deviation.

Figure 14A shows Western blotting data of the effects of FXa, EDX, ALK5-I, and EDX + ALK5-I on the production of IL-6, TGFβ1, and β-actin in B16 cells. The band densities of IL-6, TGFβ1, and β-actin on the membrane were measured; the IL-6/β-actin ratios for each group are shown in Fig. 14B , and the TGFβ1/β-actin ratios are shown in Fig. 14C . The IL-6/β-actin ratio was significantly ( *p* < 0.01) decreased by approximately 20% in the FXa + EDX and FXa + EDX + ALK5-I groups compared with the FXa group (control), whereas no decrease was observed in the FXa + ALK5-I group. These results suggest that IL-6 production in B16 cells is suppressed by inhibition of the FXa-PAR2 pathway by EDX treatment, but not by inhibition of the FXa-PAR2-TGFβ pathway by ALK5-I treatment. On the other hand, the TGFβ1/β-actin ratio level was decreased by approximately 50% in the FXa + EDX and FXa + ALK5-I groups compared with the FXa group (control), and was decreased to approximately 25% in the FXa + EDX + ALK5-I group. These results suggest that TGFβ1 production in B16 cells is suppressed not only by inhibition of the FXa-PAR2 pathway by EDX treatment, but also by inhibition of the FXa-PAR2-TGFβ pathway by ALK5-I treatment.

## Discussion

Tumor metastasis is a multistep process that requires sophisticated adaptive interactions between tumor cells and target organ cells in the host. It has been reported that metastatic melanoma cells express large amounts of TF, the main initiator of the plasma coagulation protease cascade, and promote tumor metastasis in both coagulation-dependent and independent ways. 30 31 The activated blood coagulation factors, thrombin and FXa, play important roles in tumor growth and metastasis. PAR1, the major receptor for thrombin, and PAR2, the major receptor for FXa, are widely and abundantly expressed in tumor cells and host tissue cells, and contribute to tumor cell motility and metastasis. 32

Thrombin induces tumor cell proliferation, metastasis, and angiogenesis, 33 enhances tumor cell adhesion and metastatic properties by increasing integrin αIIβIII expression in tumor cells, 34 and facilitates cell invasion 35 and EMT induction via PAR1 activation. 36 Furthermore, FXa alone, factor VIIa–FXa complex, 37 and TF–factor VIIa–FXa complex 38 promote breast cancer cell migration and invasion via PAR2 activation. Recently, we reported that among orally administered DOACs, the FXa inhibitor EDX strongly inhibited PAR2-mediated coagulation and inflammation, as well as cancer cell proliferation, and promoted apoptosis in tumor-bearing host mice. 17

In this study, we analyzed the effects of orally administered DOACs (DABE, RVX, and EDX) on metastasis of metastatic melanoma B16 cells derived from C57BL/6j mouse tumor cells implanted in female C57BL/6j mice. All DOACs suppressed the increase in B16 cell clusters in the lung and other tissues, and the increase in inflammatory markers in plasma, in saline-treated mice implanted with B16 cells. Among these DOACs, EDX showed the strongest inhibitory activity, and the effect of EDX in reducing the number of DOPA-stained cells was observed in various organs, including the lung, liver, colon, small intestine, ovary, and mesenteric lymph node, presumably through the suppression of the PAR2 pathway (data other than for the lung are not shown).

Previous studies have shown that activation of PAR2 induces cancer progression in host mice in a TGFβ signal-dependent manner. 28 In addition, it has been reported that PAR2 enhances the expression of TGFβR I (ALK5). 29 Therefore, in this study, we analyzed the effect of EDX administration on the metastasis of B16 cells transplanted into mice, mainly by analyzing the expression of PAR2 and TGFβ-related factors in plasma or lung tissue.

First, we measured factors (IL-6 and TGFβ1) in plasma, cell membrane receptors (PAR1, PAR2, TGF-β, and TGFβR I), and intracellular signaling factors (SMAD2, SMAD3, and SMAD4) in lung tissues believed to be related to cancer cell metastasis in the lungs. The results showed that all factors increased in tumor-bearing mice were significantly decreased in EDX-administered mice, except for PAR1, suggesting that EDX inhibits both the PAR2 and TGFβ pathways.

We then analyzed changes in MMP-2 and MMP-9 levels in plasma, and changes in periostin, angiopoietin-2, L-selectin, VEGFA, bFGF, and PDGF levels in lung tissues, which are angiogenesis-related factors derived from tumor cells and their surrounding cells and may be involved in B16 cell metastasis. The results showed that all factors were significantly increased in tumor-bearing mice and significantly decreased in EDX-administered mice, suggesting that EDX suppresses angiogenesis in the tissue surrounding the tumor.

Successively, the expression of angiogenesis-related factor receptors (TM, C-X-C motif chemokine receptor 1 (CXCR1), and VEGFR1), cell adhesion and migration-related molecules (E-selectin and Robo4), and intercellular tight junction molecules (claudin 5 and E-cadherin), expressed in vascular endothelial cells, was analyzed. The expression of angiogenesis-related factor receptors and cell adhesion/migration-related molecules, which was significantly increased in melanoma-implanted mice, was significantly decreased in EDX-treated mice. In contrast, the expression of intercellular tight junction molecules was significantly increased in EDX-administered mice, suggesting that EDX suppresses inflammation in vascular endothelial cells, strengthens intercellular junctions, and inhibits angiogenesis.

Furthermore, we measured the expression of EMT-related factors that was believed to be related to tumor invasion and metastasis in lung tissues. The results showed that the expression of vimentin, fibronectin, Snail-1, Wnt3a, β-catenin, and ZEB1, which was significantly increased in the lung tissue of tumor-bearing mice, was significantly decreased in EDX-administered mice, suggesting that EDX strongly suppresses EMT activity.

To clarify the type of TAM involved in PAR-2-mediated B16 cell metastasis in mice, we analyzed changes in molecular markers specific to M1- or M2-type macrophages. The results suggested that M2 macrophages are involved in melanoma metastasis in B16 cell-implanted mice and indicated the metastasis-suppressing effect of EDX.

Furthermore, to determine whether the TGFβR-mediated metastasis pathway depends on the PAR2-mediated metastasis pathway in B16 cell metastasis, we compared the effect of ALK5-I, which inhibits TGFβR I and TGFβR II complex formation, in B16 cell metastasis with that of EDX. The results indicated that both the FXa-PAR2 and FXa-PAR2-TGFβ pathways are involved in the lung metastasis of B16 cells and the induction of IL-6-mediated inflammatory injury in B16 cell-implanted mice, suggesting that EDX suppresses melanoma metastasis by suppressing both pathways through PAR2 pathway inhibition.

Finally, to clarify whether the action of EDX depends on the presence of FXa, we investigated the effects of FXa, EDX, and ALK5-I on the production of melanin, IL-6, and TGFβ1 in B16 cells cultured in vitro. The results showed that FXa promotes melanin synthesis, and that EDX and ALK5-I additively inhibit FXa-induced melanin production, suggesting that both the FXa-PAR2 and FXa-PAR2-TGFβ pathways are involved in melanin production in melanoma cells. It remains unclear whether melanomas that actively produce melanin pigments have high proliferation or tissue-invasive potential, and how inhibition of the FXa-PAR2 or FXa-PAR2-TGFβ pathways by EDX or ALK5-I treatment affects the metastatic ability of melanoma cells remains a topic for future investigation. Furthermore, western blotting analysis revealed that FXa-dependent IL-6 production was significantly inhibited by EDX alone, indicating that IL-6 production is dependent on the FXa-PAR2 pathway. On the other hand, FXa-dependent TGFβ1 production was additively inhibited by EDX and ALK5-I, suggesting that TGFβ1 production is dependent on both the FXa-PAR2 and FXa-PAR2-TGFβ pathways, similar to melanoma cell metastasis in host animals.

**Fig. 15:**
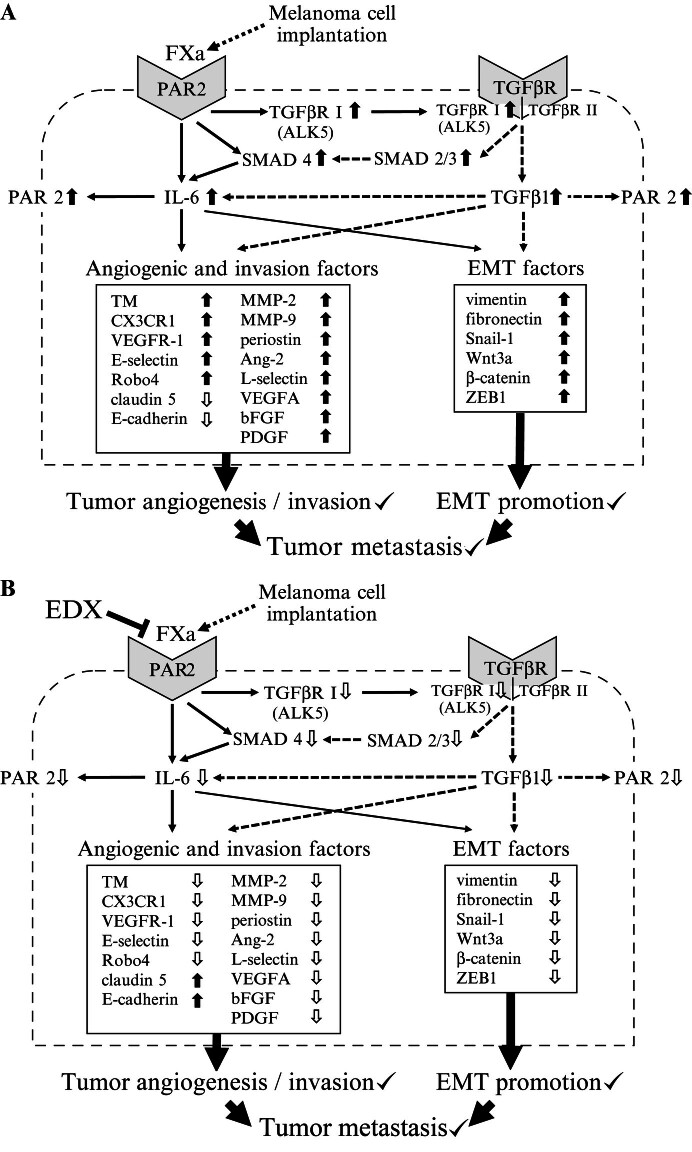
Possible mechanism of melanoma metastasis mediated by FXa-PAR2 and FXa-PAR2-TGFβ pathways in mice implanted with B16 melanoma cells. ( **A** ) In the absence of EDX, FXa produced by activation of the blood coagulation system and/or cancer-associated FXa-like protease in hosts implanted with melanoma cells activates the FXa-PAR2 and FXa-PAR2-TGFβ pathways in living tissue and melanoma cells, resulting in a decrease in tight junction-associated factors (claudin 5 and E-cadherin) between tissue cells, and an increase in other tumor-associated factors, thereby promoting inflammation, tumor angiogenesis, tissue invasion, and EMT in the host body, and promoting metastasis of melanoma cells. ( **B** ) In mice treated with EDX, activation of the FXa-PAR2 and FXa-PAR2-TGFβ pathways is suppressed, tight junction-associated factors are increased, and other angiogenic, invasion, and EMT-associated factors are decreased, resulting in the suppression of inflammation, angiogenesis, tissue invasion, and EMT, and the inhibition of melanoma cell metastasis. Ang-2, angiopoietin-2; bFGF, basic fibroblast growth factor; EDX, edoxaban; EMT, epithelial–mesenchymal transition; FXa, factor Xa; IL-6, interleukin 6; MMP, matrix metalloproteinase; PAR, protease-activated receptor; PDGF, platelet-derived growth factor; SMAD, small mothers against decapentaplegic; Snail-1, small family zinc finger 1; TGFβ1, transforming growth factor β1; TGFβR I, TGFβ receptor type I; TGFβR II, TGFβR type II; VEGFA, vascular endothelial growth factor A; Wnt3a, wingless MMTV integration site family, member 3a; ZEB1, zinc finger E-box binding homeobox 1.

Based on these results, we speculated on the mechanisms underlying the involvement of the FXa-PAR2 and FXa-PAR2-TGFβ pathways in the metastasis of mouse melanoma cells implanted with B16 cells, as well as on the inhibitory mechanism underlying EDX treatment. In the absence of EDX treatment ( Fig. 15A ), FXa produced by activation of the coagulation system and/or cancer-associated FXa-like protease in the host body due to implantation of melanoma cells activates the FXa-PAR2-TGFβ pathway in living tissue and melanoma cells, reduces tight junction-associated factors (claudin 5 and E-cadherin) between tissue cells, and increases other tumor-associated factors. This promotes inflammation, tumor angiogenesis, tissue invasion, and EMT in the host body, and promotes metastasis of melanoma cells. On the other hand, in mice administered EDX ( Fig. 15B ), activation of the FXa-PAR2 and FXa-PAR2-TGFβ pathways is suppressed, tight junction-associated factors are increased, and other factors are decreased. Thus, inflammation, angiogenesis, tissue invasion, and EMT are suppressed, and melanoma cell metastasis is inhibited. According to this hypothesis, administration of EDX inhibits FXa produced in the host body and FXa-like proteases derived from cancer cells, thereby suppressing the metastasis of melanoma cells through PAR2 activation.

This study also demonstrated that both DABE and RVX significantly inhibited melanoma metastasis and decreased plasma IL-6 and sTM levels; however, their inhibitory effects were significantly weaker than those of EDX. Considering that PAR1 is required for experimental lung metastasis of thrombin-stimulated cells, 39 and that the interactions between PAR1, PAR2, and TGFβ signaling factors are involved in matrix formation, which promotes tumorigenesis, 40 DABE may have suppressed tumor metastasis and inflammation by inhibiting the thrombin-PAR1 pathway, subsequently affecting the signaling factors involved in the PAR2 and TGFβ pathways. The exact mechanism by which DABE inhibits metastasis requires further analysis.

RVX inhibition of B16 cell metastasis is likely mediated by PAR2, similar to EDX. However, the reason why EDX was more effective than RVX remains unclear. In our previous study on the effect of DOACs on the proliferation of Colon26 cells, EDX also showed a stronger effect than RVX, similar to the results of the current study. The reason for this, as hypothesized in our previous paper, 17 is likely that in human pharmacokinetics studies, the *T*_max_ value (the time until the drug reaches its maximum concentration in the blood after administration) of EDX is higher than that of RVX.

Among the known proteases of the blood coagulation and fibrinolysis systems, such as thrombin, FXa, factor VIIa, factor IXa, factor XIa, tissue plasminogen activator, plasmin, activated protein C, trypsin, and chymotrypsin, EDX has been reported to specifically inhibit FXa. 41 However, it remains possible that EDX may be more effective than RVX in inhibiting tumor cell-derived FXa-like proteases, which are different from the FXa of the blood coagulation system involved in tumor metastasis.

The results of this study suggest that EDX therapy may not only prevent recurrent thrombus formation in cancer patients with VTE but may also have the effect of suppressing the proliferation and metastasis of cancer tissue cells. Further basic and clinical research is needed to clarify the inhibitory effects of DOACs, including EDX, on cancer cell proliferation and metastasis.

## Conclusion

Among the DOACs tested in this study, EDX showed the most significant inhibitory effect on B16 cell metastasis in mice. Administration of EDX suppressed the increase in inflammatory factors, angiogenesis/invasion-related factors, and EMT-related factors in the lungs of mice implanted with B16 cells. Furthermore, EDX inhibited the FXa-dependent production of melanin, IL-6, and TGFβ1 in cultured B16 cells. These results suggest that EDX may inhibit inflammation, angiogenesis, and EMT in tumor cells and surrounding stromal cells by inhibiting FXa-dependent PAR2 and TGFβ signaling pathways, thereby suppressing B16 cell metastasis.

## Notes

**What Is Known about This Topic?**

- Direct oral anticoagulants (DOACs), such as dabigatran etexilate, rivaroxaban, and edoxaban (EDX), inhibit the growth of Colon26 tumor cells derived from syngeneic mice when implanted into BALB/c mice. In particular, the factor Xa-specific EDX shows a high efficacy.
- EDX suppresses hypercoagulability and enhanced inflammatory responses in tissues of tumor-bearing mice via the factor Xa-PAR2 pathway, inhibits tumor cell proliferation, and induces tumor cell death (apoptosis).

**What Does This Paper Add?**

- All the DOACs tested (dabigatran etexilate, rivaroxaban, and EDX) inhibited the metastasis of syngeneic mouse melanoma B16 cells implanted in female C57BL/6J mice. Among them, EDX showed the most potent anti-metastatic effect.
- EDX inhibits melanoma cell metastasis by suppressing inflammation, angiogenesis, and epithelial-mesenchymal transition in melanoma cell-implanted mice. Furthermore, EDX inhibited factor Xa-dependent production of melanin, IL-6, and TGFβ1 in B16 cells in vitro.
- The mechanism by which EDX inhibits metastasis in B16 cell is suggested to involve the inhibition of factor Xa-dependent PAR2 and TGFβ signaling pathways in both tumor cells and surrounding tissue cells.
